# Complete Coding Sequence of Usutu Virus Strain Gracula religiosa/U1609393/Belgium/2016 Obtained from the Brain Tissue of an Infected Captive Common Hill Myna (*Gracula religiosa*)

**DOI:** 10.1128/genomeA.00042-17

**Published:** 2017-03-23

**Authors:** Steven Van Borm, Bénédicte Lambrecht, Frank Vandenbussche, Mieke Steensels

**Affiliations:** a Molecular Platform, Veterinary and Agrochemical Research Center, Ukkel, Belgium; b Avian Virology and Immunology, Veterinary and Agrochemical Research Center, Ukkel, Belgium

## Abstract

The complete and annotated coding sequence and partial noncoding sequence of an Usutu virus genome were sequenced from RNA extracted from a clinical brain tissue sample obtained from a common hill myna (*Gracula religiosa*), demonstrating close homology with Usutu viruses circulating in Europe.

## GENOME ANNOUNCEMENT

Usutu virus is a mosquito-borne flavivirus belonging to the Japanese encephalitis virus serocomplex. Originally discovered in South Africa in 1959 (1), it was first demonstrated in Europe in 2001 with a die-off of Eurasian blackbirds (*Turdus merula*) and great grey owls (*Strix nebulosa*) in Austria (2). The virus subsequently expanded throughout Europe, causing unusually high levels of mortality in blackbirds (3).

In October 2016, a brain tissue sample from a captive common hill myna (*Gracula religiosa*) that died 24 h after the first demonstration of clinical symptoms tested positive using an in-house Usutu virus real-time reverse transcription-PCR (RT-PCR) (data not shown). During autopsy, a swollen liver and spleen were detected as sole macroscopic lesions. The brain tissue was homogenized in phosphate-buffered saline (10% [wt/vol]), pretreated by 0.45-µM-pore-size selective filtration and nuclease, and RNA was extracted as previously described (4, 5). cDNA was synthesized using SuperScript IV reverse transcriptase (Thermo Fisher Scientific) and random hexamer primers, according to the manufacturer’s instructions, followed by double-strand cDNA synthesis using the NEBNext mRNA second-strand synthesis module (New England BioLabs), according to the manufacturer’s instructions. Sequencing libraries were prepared starting from 1 ng (or the maximum amount available) of cDNA using the Nextera XT kit (Illumina), according to the manufacturer’s instructions, quantified with the library quantification kit Illumina platforms (Kapa Biosystems), and fragment length distribution was verified using the Bioanalyzer with the high-sensitivity DNA kit (Agilent Technologies). Sequencing was performed using a MiSeq reagent kit version 3 (Illumina) with 2 × 300-bp paired-end sequencing. Thirteen libraries were multiplexed using standard Illumina indexing primers.

The quality of the sequences was checked with the FastQC tool version 0.10.1 (http://www.bioinformatics.babraham.ac.uk/projects/fastqc/). Stretches containing unidentified nucleotides (N) were trimmed using Cutadapt version 1.3 (6) prior to quality trimming using Sickle version 1.210 (*Q* score <30, length <50 bp) (7). *De novo* assembly was performed using MIRA version 4.0.2, with default settings (http://mira-assembler.sourceforge.net/) (8). The protein coding sequences were predicted relative to the sequence with accession no. KJ438768 using GATU (9).

The nearly complete genome of Usutu virus strain Gracula religiosa/U1609393/Belgium/2016 was obtained with an average coverage of 493× and contains a single 10,305-nucleotide (nt) open reading frame (ORF) encoding a polypeptide precursor protein, sharing a high nucleotide homology with Usutu viruses circulating in wild birds and mosquitoes in Germany in 2011 to 2013 and Usutu viruses circulating in 2011 to 2015 in central and western Europe in general.

### Accession number(s).

The complete coding sequence of Usutu virus strain Gracula religiosa/U1609393/Belgium/2016 was assigned DDBJ/EMBL/GenBank accession no. KY315178.
